# Twin‐twin transfusion syndrome with anemia‐polycythemia developed during the third trimester of pregnancy

**DOI:** 10.1002/ccr3.3609

**Published:** 2020-12-04

**Authors:** Tatsunori Shiraishi, Osamu Kizumi, Sakae Kumasaka, Shunji Suzuki

**Affiliations:** ^1^ Department of Obstetrics and Gynecology Japanese Red Cross Katsushika Maternity Hospital Tokyo Japan

**Keywords:** anemia‐polycythemia, third trimester of pregnancy, twin‐twin transfusion syndrome

## Abstract

We present here a rare case of twin‐twin transfusion syndrome with anemia‐polycythemia developed at 34‐35 weeks of gestation.

## Graphical abstract

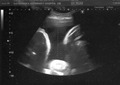

## 1 INTRODUCTION

Some case series of twin‐twin transfusion syndrome (TTTS) with anemia‐polycythemia (AP) prior to laser surgery performed at 16‐25 weeks of gestation have been reported; however, we present here a case of TTTS with AP developed at 34‐35 weeks of gestation.

Unbalanced feto‐fetal blood flow in monochorionic twin pregnancy may lead to twin‐twin transfusion syndrome (TTTS) or twin anemia‐polycythemia sequence (TAPS).^1^, ^2^ TTTS and TAPS have been described as two mutually different entities. TTTS arises from an unbalanced net blood flow from donor to recipient through various large placental anastomoses, resulting in large amniotic fluid discordances between the twins, while TAPS arises from an unbalanced and chronic net transfusion through only a few minuscule (diameter <1 mm) vascular anastomoses. To date, although some case series of TTTS with anemia‐polycythemia (AP) prior to laser surgery performed at 16‐25 weeks of gestation have been reported,^1^, ^3^, ^4^ cases of co‐existing AP in twin pregnancy with TTTS may be rare during the third trimester. However, we present here a case of TTTS with AP developed at 34‐35 weeks of gestation.

## 2 CASE REPORT

**FIGURE 1:**
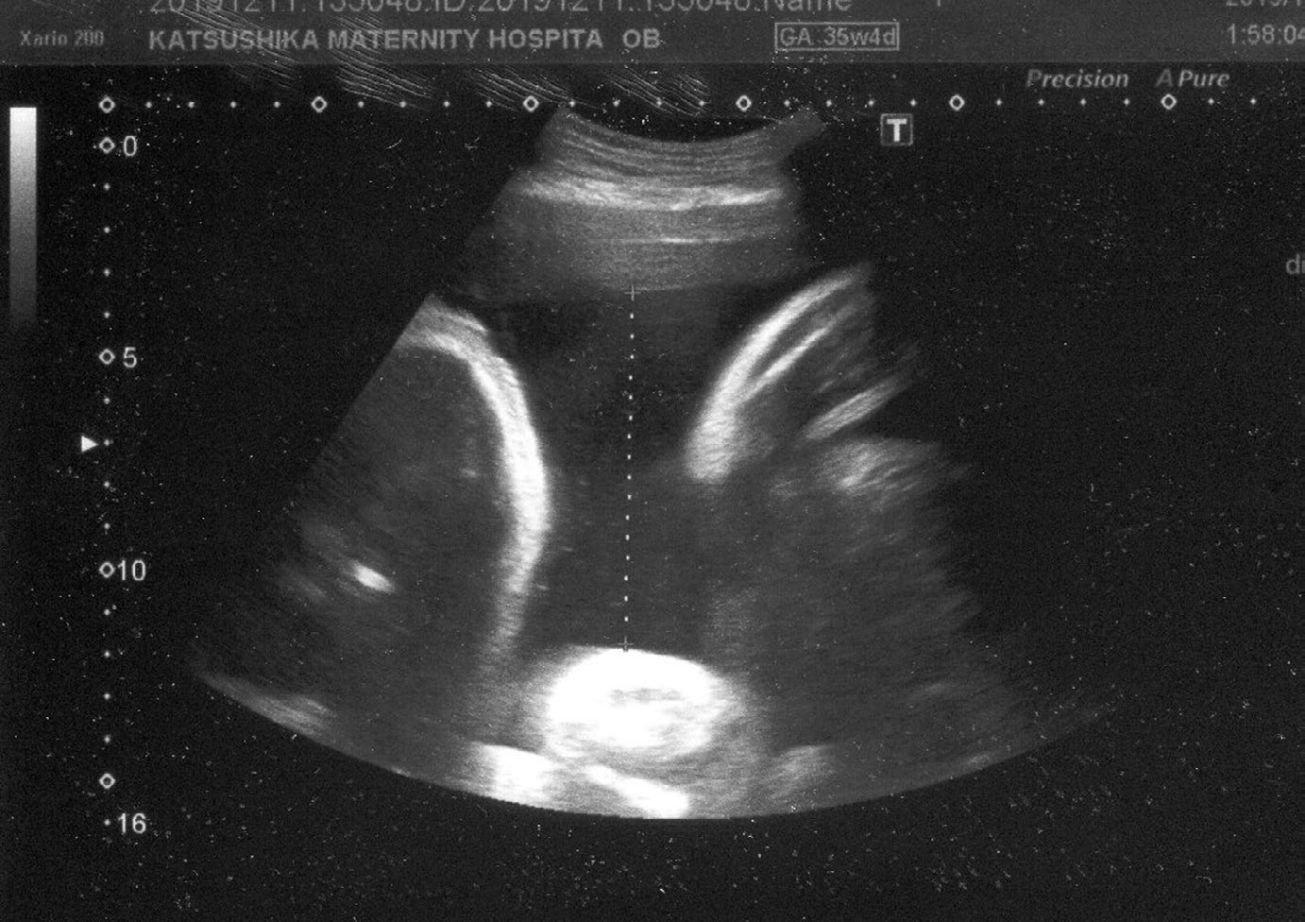
Amniotic pocket of Twin A at 35 wk and 4 d of gestation

**FIGURE 2:**
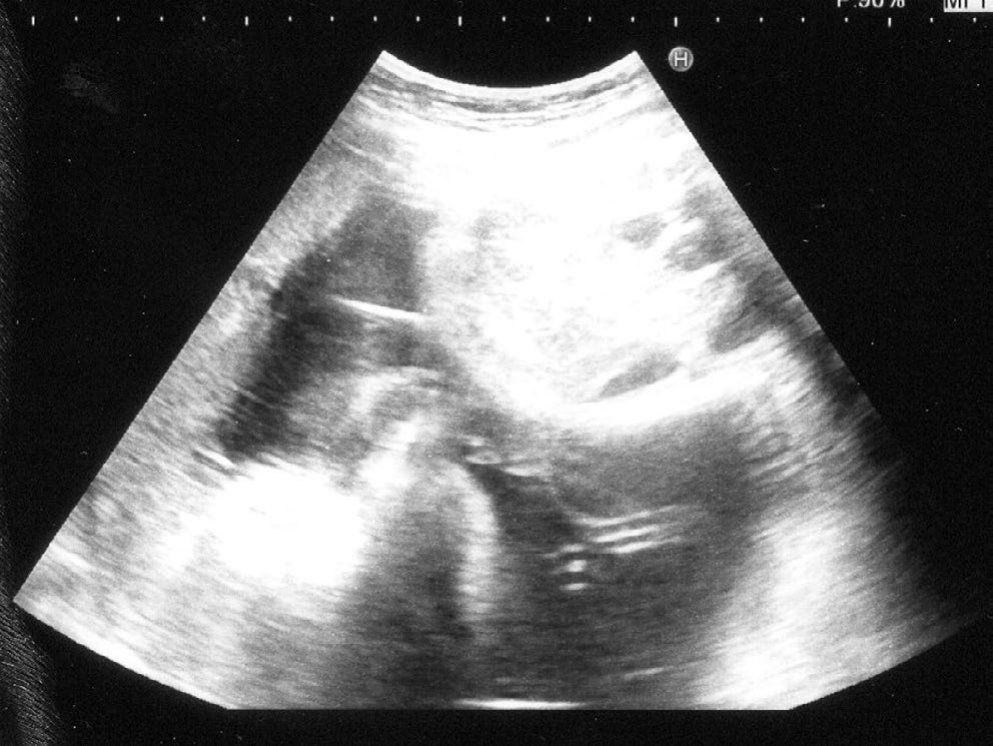
Amniotic fluid space of twin B at 35 wk and 4 d of gestation (upper space)

**FIGURE 3:**
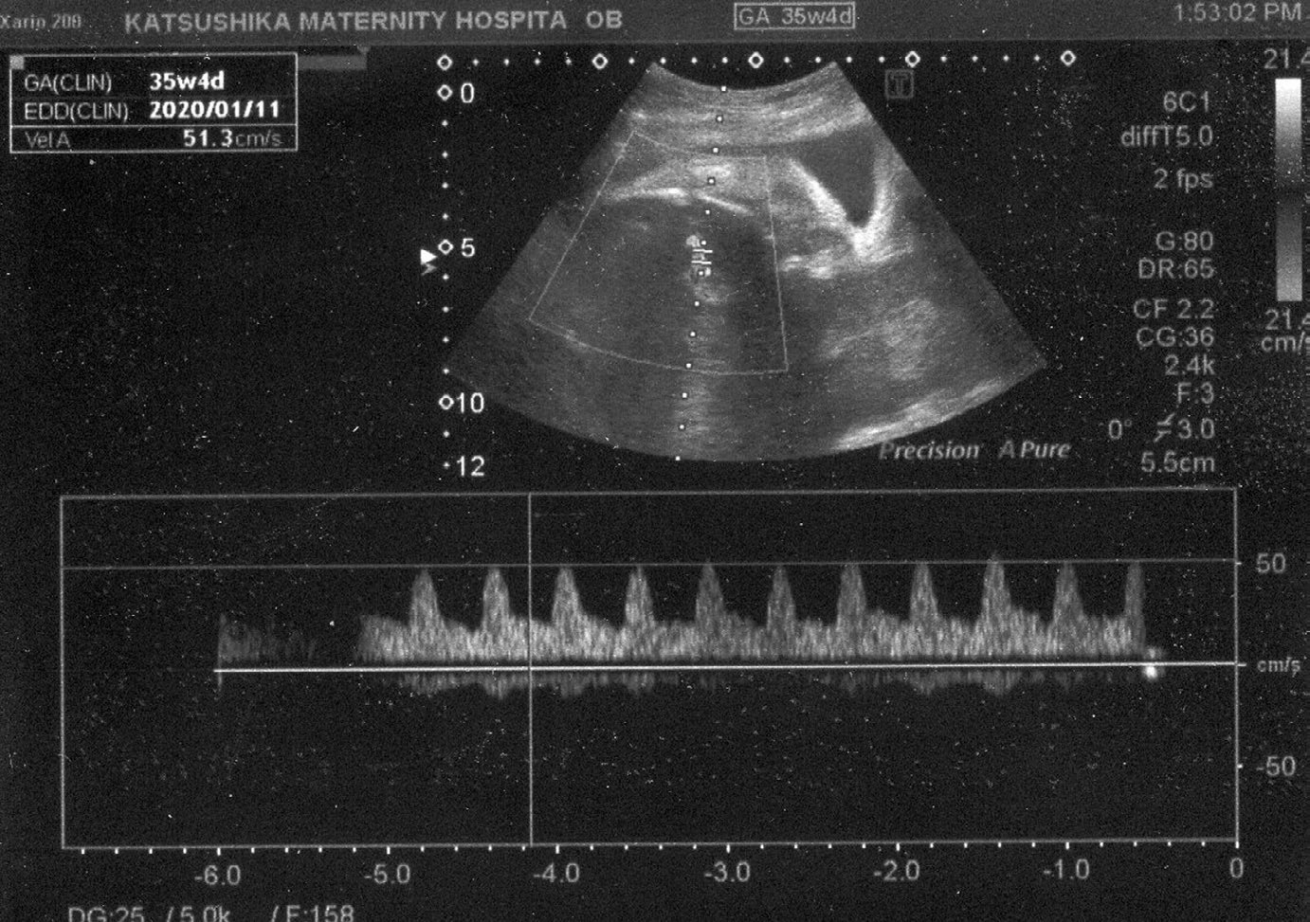
Fetal middle cerebral artery flow in twin A at 35 wk and 4 d of gestation

**FIGURE 4:**
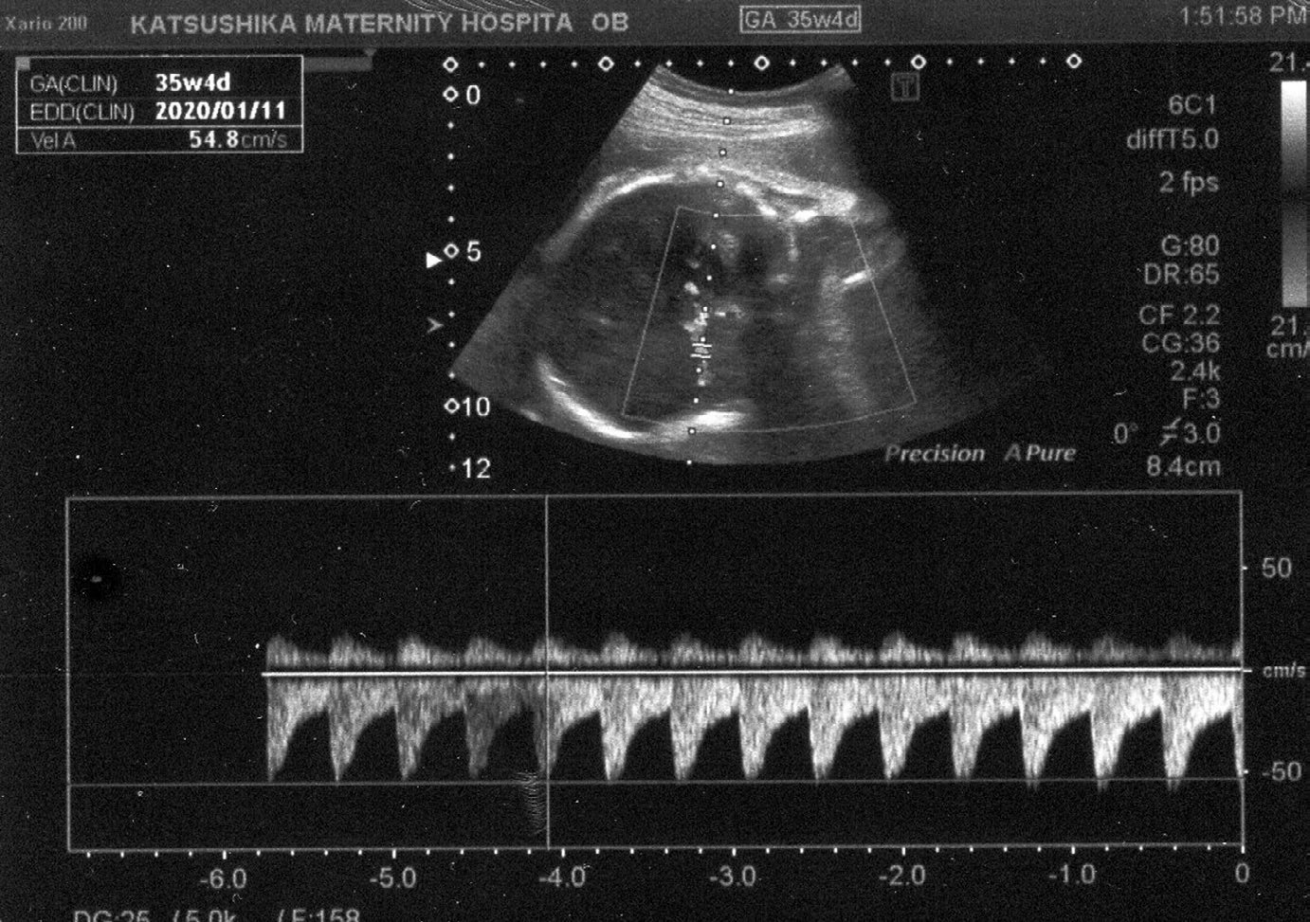
Fetal middle cerebral artery flow in twin B at 35 wk and 4 d of gestation

**FIGURE 5:**
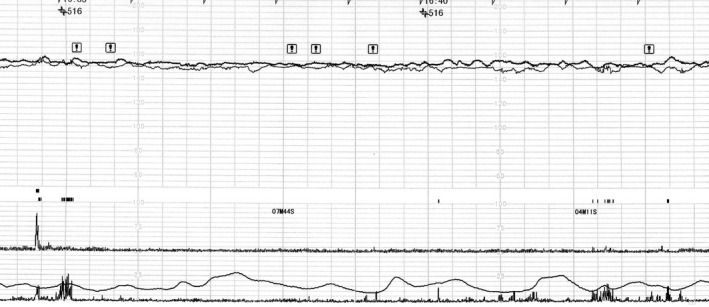
Fetal heart rate tracings showing the decreased baseline variability in the both twins at 35 wk of gestation

A 41‐year‐old gravida 2 para 1 was referred to our hospital because of monochorionic‐diamniotic (MD) twin pregnancy. Her MD twin pregnancy had progressed uneventfully until 34 weeks of gestation based on weekly ultrasonic examinations. At 34 weeks and 4 days of gestation, the amniotic pockets (APs) and the fetal middle cerebral artery peak systolic velocities (MCA‐PSVs) of twin A and B were normal (4.9 and 3.8 cm, and 47.1 and 53.4 cm/sec; 1.5 MoM = 73 cm/sec) by an ultrasound examination. At 35 weeks and 4 days of gestation, however, the APs of twin A and B were 8.2 and 1.9 cm (Figures 1 and 2), respectively. The fetal MCA‐PSVs of twin A and B were normal (51.3 and 54.8 cm/sec, Figures 3 and 4; 1.5 MoM = 77 cm/sec); however, the fetal heart rate tracings showed the decreased baseline variability in the both twins without apparent uterine contractions (Figure 5). A cesarean section was performed at the same day because of twin‐twin transfusion syndrome (TTTS) stage 1 with nonreassuring fetal status. At delivery, twin A was a 1670‐g male infant with Apgar scores of 8 and 9 at 1 and 5 minutes, respectively, while twin B was a 1764‐g male with Apgar scores of 8 and 9, respectively. The hemoglobin concentration of twin A was 27.7 g/dL (normal: 13‐22 g/dL) with reticulocyte counts of 6.0% (normal: <7%), while it was 10.4 g/dL with reticulocyte counts of 6.1% in twin B. After delivery, twin A required intravenous infusion of 4.4% human serum albumin for correction of polycythemia, while twin B required a transfusion of red cell concentrate (61 mL). The placenta was confirmed as monochorionic without superficial arterio‐arterial (AA) or veno‐venous (VV) anastomoses by milk injection. There were no color differences in the placenta.

## 3 DISCUSSION

Cases with large intertwin hemoglobin (Hb) differences of AP can be classified as chronic or acute onset.^5^ In addition to TAPS leading to a slow and chronic intertwin blood transfusion, acute TTTS occurring by an acute intertwin blood transfusion as a result of uterine contractions can be mentioned. In the current case, there were not significant differences in the neonatal reticulocyte counts or color in the placenta indicating the presence of TAPS although the influences of TTTS on them are not clear.^2^ The development of acute TTTS is thought to be mainly mediated by large and superficial AA or VV anastomoses, since these anastomoses have a low resistance to allow blood to flow rapidly from one twin to the other.^5^, ^6^ We could not find the presence of AA or VV anastomoses in the placenta; however, the AP is presumed to be caused by a more rapid blood shift than that of TAPS. Unfortunately, we could not provide a placental photograph in the report. However, the current case may be very rare. In the current study, any apparent uterine contractions were not recorded on the cardiotocogram; however, the uterine contraction might have occurred in association with rapid development of polyhydramnios. The contraction might have caused a more rapid blood shift than that of TTTS via arterio‐venous anastomoses in the placenta. Otherwise, acute polyhydramnios may have resulted in rapid compression in the amniotic sac leading to fetal heart load and/or hypotension associated with rapid blood inflow.^7^, ^8^ They may have led to rapid intertwin transfer of blood the patent anastomoses.

## 4 CONCLUSION

We encountered a case of TTTS with AP developed during the third trimester of pregnancy.

## CONFLICT OF INTEREST

All authors declare no conflict of interest relevant to this article.

## AUTHOR CONTRIBUTIONS

TS (Primary author): analyzed the data and wrote the manuscript. KO and KS (Primary care physicians): analyzed the data. SS: involved in the idea of the manuscript, analyzed the data, and drafted the manuscript.

## ETHICS APPROVAL AND CONSENT TO PARTICIPATE

The protocol for this case report was approved by the Ethical Committee of the Japanese Red Cross Katsushika Maternity Hospital (Shunji Suzuki: K1918). Written informed consent for publication of the clinical details as obtained from the parent of the patient study.
